# An mHealth Platform for Supporting Clinical Data Integration into Augmentative and Alternative Communication Service Delivery: User-Centered Design and Usability Evaluation

**DOI:** 10.2196/rehab.9009

**Published:** 2018-07-24

**Authors:** Erh-Hsuan Wang, Leming Zhou, Szu-Han Kay Chen, Katya Hill, Bambang Parmanto

**Affiliations:** ^1^ School of Pharmacy University of Pittsburgh Pittsburgh, PA United States; ^2^ Department of Health Information Management University of Pittsburgh Pittsburgh, PA United States; ^3^ Department of Communication Disorders and Sciences State University of New York at Fredonia Fredonia, NY United States; ^4^ Department of Communication Science and Disorders University of Pittsburgh Pittsburgh, PA United States

**Keywords:** Web-based portal, data integration, Augmentative and Alternative Communication, service delivery

## Abstract

**Background:**

The recent trend of increasing health care costs in the United States is likely not sustainable. To make health care more economically sustainable, attention must be directed toward improving the quality while simultaneously reducing the cost of health care. One of the recommended approaches to provide better care at a lower cost is to develop high-quality data collection and reporting systems, which support health care professionals in making optimal clinical decisions based on solid, extensive evidence.

**Objective:**

The objective of this project was to develop an integrated mobile health Augmentative and Alternative Communication (AAC) platform consisting of an AAC mobile app and a Web-based clinician portal for supporting evidence-based clinical service delivery.

**Methods:**

A questionnaire and interviews were used to collect clinicians’ ideas regarding what constitutes their desired “clinically relevant” data. In response, a Web-based portal was designed by combining mobile and Web technologies with an AAC intervention to create an integrated platform for supporting data collection, integration, and reporting. Finally, a usability study was conducted with health care professionals.

**Results:**

A Web-based portal was created and integrated with a tablet-based AAC mobile app and data analysis procedures. In the usability study, all participants agreed that the integrated platform provided the ability to collect comprehensive clinical evidence, automatically analyze collected data in real time, and generate clinically relevant performance measures through an easily accessible Web-based portal.

**Conclusions:**

The integrated platform offers a better approach for clinical data reporting and analytics. Additionally, the platform streamlines the workflow of AAC clinical service delivery.

## Introduction

Improving health care quality while simultaneously reducing costs is a challenging task. To achieve these goals simultaneously, the Institute of Medicine (IOM) recommends the use of information technologies for capturing clinical data that may be then integrated into the process of clinical decision making during care delivery [1]. According to this recommendation, health care professionals are required to collect, analyze, report, and review their patients’ performance data before adjusting treatments. This might be a rather onerous task, given the already demanding nature of health care professions.

Mobile technologies, however, have offered those in health care professions myriad new and low-effort ways of adhering to IOM’s recommendations, especially with regards to capturing patient data. Growing evidence has demonstrated that mobile health (mHealth) platforms make it possible for health care professionals to monitor patient conditions constantly and remotely. Furthermore, mHealth platforms can empower patients to manage their own diseases [2-12]. The integration of mobile technologies into health care may optimize health care practices by enabling professionals to conveniently collect and utilize large amounts of patient data in their clinical practice [13,14]. In this model, convenient data collection, integration, and reporting is of critical importance.

Communication is essential for maximizing quality of life. It is estimated that approximately 10% of the US adult population reported a communication disability, temporary or permanent, because of various reasons, such as hearing loss, head injury, stroke, autism, dementia, cancer, vocal problems, intellectual disability, and neurological causes [15]. Some of these patients use the service offered by Augmentative and Alternative Communication (AAC) technologies, which can enable them to communicate with others and improve their language abilities.

The information technology–supported data collection and integration is especially important within the field of AAC in terms of optimizing health care practices. In AAC, it is necessary to collect, evaluate, and integrate the best clinical evidence available for decision making such as patient assessment and treatment plan adjustment [16,17]. Currently, there are two data collection and reporting approaches in AAC. The first approach has been specifically designed for dedicated AAC devices [18]. In this approach, language samples are stored in the AAC device as text files. For analysis, speech-language pathologists (SLPs) need to retrieve this text file using a USB flash drive from patients’ AAC device when they visit the clinic and load the text file into a specially designed program, where each text item in the file must be manually converted into individual utterances by SLPs. Eventually, the program processes these utterances and generates a language performance report. Evidently, this is a very labor-intensive procedure, and many SLPs choose to forego this data analysis—and, thus, the performance report—altogether. Without the resultant performance reports available when the patient visits the clinic, the adjustment of the patient’s treatment strategy may be delayed.

The second data collection and reporting approach has been implemented in some mobile AAC apps [19]. These AAC apps may collect data, generate performance reports, and provide SLPs a brief summary of patient performance along with a full word or utterance list. From here, SLPs can either review the results directly in the app or ask their patients (or caregivers of their patients) to email the report through the app. These data items, however, are not collected according to any research studies that might indicate the needs of SLPs, and, therefore, they are of limited clinical value.

The objective of this project was to propose and evaluate a solution to the problems existing in these two currently available data collection and reporting approaches in AAC. More specifically, the collected data should be clinically relevant and the data as well as the reports generated from the data should be readily available to SLPs when needed.

Indeed, the portability of mobile apps is critical in facilitating real-time patient data collection and reporting this data to their users. A Web-based portal can further augment the data reporting process to health care providers. Such portals might serve as widely accessible resources for health care professionals in conveniently accessing their patients’ clinical performances from anywhere and at any time. Taken together, mobile apps and a Web-based portal form an effective platform for providing AAC services and allowing SLPs and persons with communication disabilities (PwCDs) to pursue dynamic treatment options.

This paper presents the development and evaluation of an mHealth platform capable of facilitating treatment in the abovementioned dynamic manner. To this end, we implemented a mixed method for identifying clinicians’ requirements on the mHealth platform as well as clinically relevant data items, which include literature review, interview, and questionnaire studies. Additionally, we conducted a study in tandem with health care professionals to evaluate the usability of this mHealth platform and to differentiate the preferences among three different approaches for patient data collection and reporting. This mHealth platform will make data collection and reporting in AAC service delivery easy and efficient if implemented as expected.

## Methods

### Requirement Analysis

We aimed to create a platform for clinicians that augments the collection of patient data and the generation of clinically relevant reports. This data collection and reporting process can be optimized in the predesign phase by surveying clinicians to determine what they see as the most relevant and specific platform. For instance, we must answer questions such as the following: What current data collection approaches are being used by SLPs? What types of clinically relevant data should be collected from PwCDs when they are using an AAC device? Which data items should be stored? How is language performance measured and what specific outcomes do SLPs desire to see in result reports? If the patient information will be presented on a Web-based portal, what are the desired features?

To this end, interview questions and a questionnaire were designed and administered to two groups of SLPs. A brief summary of the interview and questionnaire studies is provided below. The details of these two studies have been previously described [20].

#### Interview With Clinicians

To better understand the needs of SLPs with regards to a Web-based clinician portal as well as their perspective on AAC patient-generated data, interviews were performed. Five SLPs were recruited from the greater Pittsburgh area through professional referrals. Each of these SLPs had 5-12 years of work experience in the field of AAC. All participants were asked a series of open-ended questions during the course of a 1-hour interview. Notably, none of the participants expressed satisfaction with current data collection approaches; furthermore, they expected a new approach capable of automatically transmitting real-time patient data to SLPs that also conveniently provides clinically relevant information.

All 5 participants expressed the belief that a Web-based clinician portal could support personalized AAC service delivery, enhance the current data collection and reporting process, and ultimately improve both the quality and efficiency of AAC-based language rehabilitation. Furthermore, these participants indicated several desired features on the Web-based clinician portal such as real-time remote monitoring, a dashboard overview, and detailed language performance information. They expected being able to select data items, collected within a certain time frame, and then flexibly export data in a variety of ways. Markedly, 80% (4/5) participants noted that tracking AAC usage time could generate a critical dataset. Moreover, 60% (3/5) participants expressed a need to distinguish between user logs generated by the patients and their family members. Further outcome measurements included communication rate, mean length of utterance, and error rate.

#### Questionnaire Study With Clinicians

A questionnaire study aimed at identifying specific information for clinical intervention and language rehabilitation outcome measurements was administered to SLPs. The questionnaire was a revised form of the AAC Sampling Procedures and Performance Monitoring Questionnaire [18], although some questions were updated because of advancements in AAC and information technologies. A total of 26 SLPs, each with 1-34 years of AAC field experience, responded. Table 1 presents the measurements believed to be important by ≥70% (14/20) of the respondents. The results of this questionnaire study were used to guide the design and development of the Web-based clinician portal, which is discussed further in the Web-based portal and data analysis section.

#### Key Characteristics of the Desired mHealth Platform

Based on the requirements collected from SLPs in the interview and questionnaire studies, we identified five essential characteristics that the new system must meet. The system must be comprehensive, automatic, in real time, clinically relevant, and easily accessible. These characteristics can be integrated in the process of data collection and reporting in AAC clinical service delivery using the mHealth platform.

**Figure 1:**
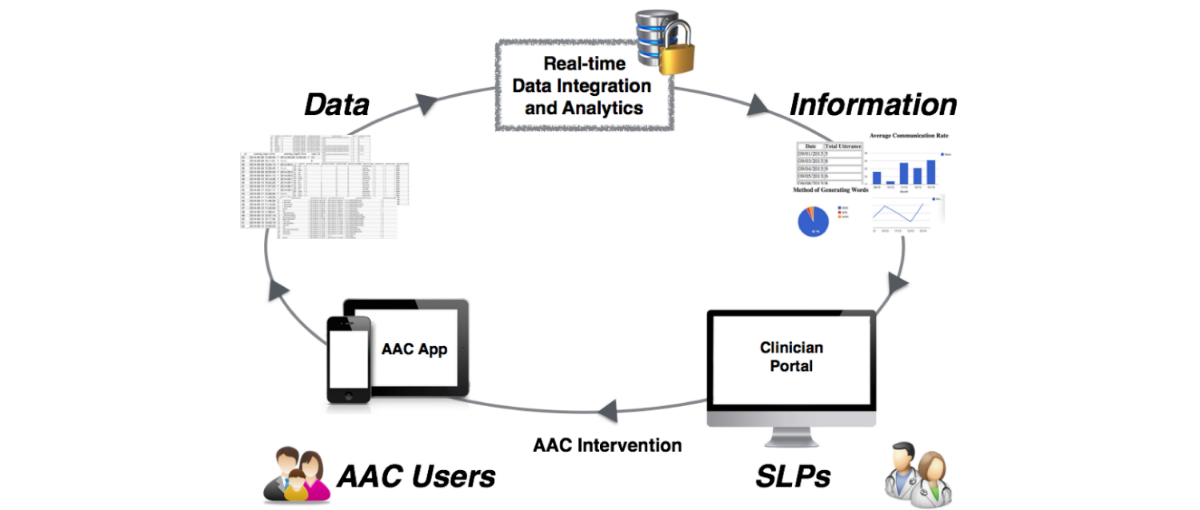
The mHealth platform for Augmentative and Alternative Communication (AAC) data collection, reporting, and clinical service delivery. SLP: speech-language pathologist.

Figure 1 illustrates the current model for the AAC mHealth platform’s data collection, integration, and reporting. Every time a user (PwCD) logs on, the mobile AAC app collects *comprehensive* language and behavioral data. This data is then transmitted, in *real time,* to a secure server, where it is *automatically* integrated and analyzed. From this patient-generated dataset, *clinically relevant* performance measurements will be obtained and then forwarded to a Web-based clinician portal, rendered in an *easily accessible* visual format, from which the clinicians (SLPs) may design and deliver personalized AAC intervention to their patients.

To create this mHealth platform, the mobile AAC app, data integration and analysis procedures, and the Web-based portal need to be built and integrated together. The mobile AAC app has already been created, and its details have been described in a previous study [21]. In this study, this app will be briefly described in the following section.

**Table 1:** Important language performance measurements indicated by the majority of respondents.

Selected summary measures	n (%)
Frequency of using the app (n=22)	20 (91)
Patient’s language performance at home (n=22)	20 (91)
Percentage of vocabulary used in the app (n=20)	18 (90)
Total number of words (n=20)	17 (85)
Utterance structures (n=22)	18 (82)
Total number of utterances (n=22)	17 (77)
Average communication rate in words (n=22)	17 (77)
Mean length of utterances in words (n=22)	16 (73)
Frequency of performing other activities in the app (AAC^a^, training, etc; n=22)	16 (73)
Total number of different words (n=20)	14 (70)

^a^AAC: Augmentative and Alternative Communication.

### EuTalk: A New Mobile Augmentative and Alternative Communication App

A mobile AAC app, EuTalk, was created to collect activity and language data from users [21]. This tablet-based AAC app was designed to incorporate the communication interface, training, and reporting capabilities. The communication interface provides both the communication program and clinical treatment exercises for PwCDs. PwCDs can use the mobile app to communicate with other people and attend trainings under the guidance of SLPs.

With EuTalk, each individual activity a user performs in the app—along with all clinically relevant language data items identified in the questionnaire and interview studies—is logged in a local SQLite database, with a corresponding timestamp, and then forwarded to a MySQL database on a secure server. Notably, the local SQLite database does not have any patient identifiers. The data stored on the local database is used to provide progress reports to the app user. Between the mobile app and the secure server, there is one randomly generated but unique number for each app user, which can be used to match the patient records on the secure server.

### Web-Based Portal and Data Analysis Procedure

A Web-based clinician portal (Figure 1) aimed at providing SLPs with detailed patient language performance information was developed and deployed on the secure server. This Web-based portal includes three major components: dashboard overview, performance reports, and administration. Since the mobile AAC app forwards all collected data to the server database in real time, this Web-based clinical portal can easily provide the raw data and processed information to SLPs, making it possible for SLPs to have easily accessed, real-time, remote monitoring of their patients’ language performance.

**Figure 2:**
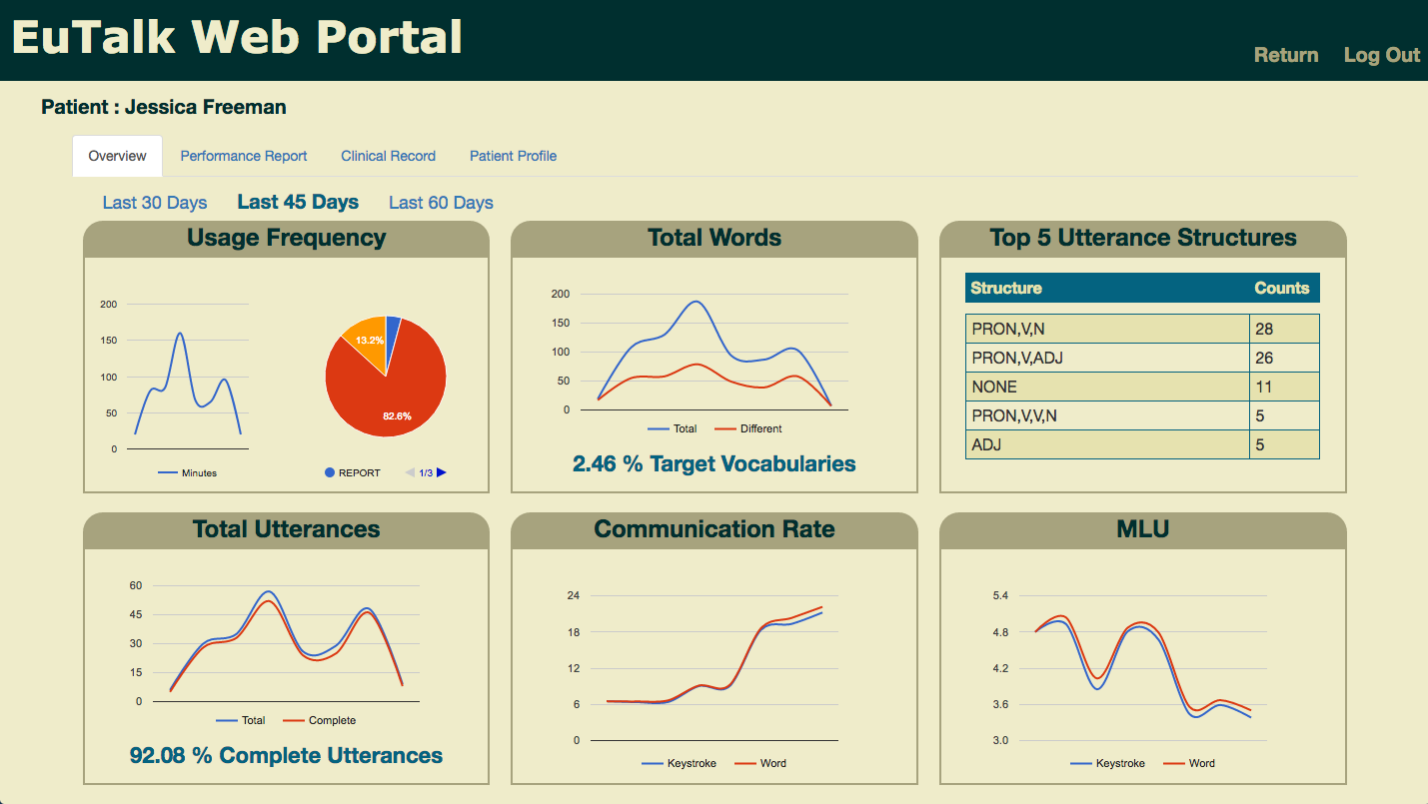
Web-based portal—dashboard overview. MLU: mean length of utterance.

The dashboard presented in Figure 2 provides a sample of what SLPs might see on a graphical interface. This particular interface allows SLPs to examine patients’ comprehensive language performance over a period of time. The specific language performance measures included in such graphics are dependent on the information identified in the questionnaire and interview studies. For instance, the dashboard in Figure 2 shows the results for the app’s frequency of use, the users’ total word count, top five utterance structures, total number of utterances, average communication rate in words, and the mean length of utterances—all presented in line charts as dependent variables with respect to time. While the default interval is 1 week, SLPs can choose to review these results in different timeframes: 30, 45, and 60 days. Additionally, the app’s frequency of use, split between the various intra-app activities, is given in a pie chart. Furthermore, the top five utterance structures are listed in a table with both the sentence structure and the number of times each structure is used. Finally, the percentage of complete utterances in all generated utterances as well as the percentage of target vocabulary used in the mobile app is shown on the dashboard.

**Figure 3:**
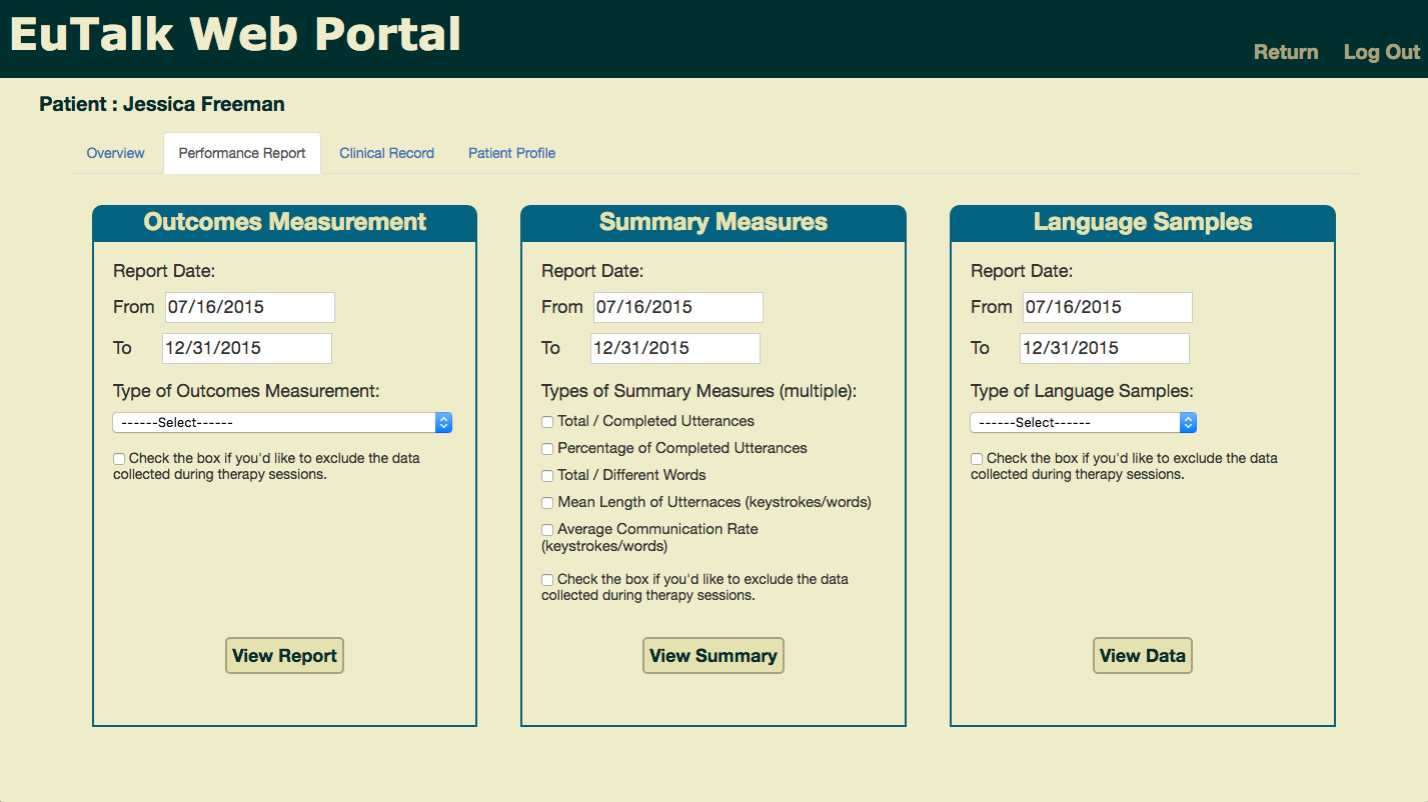
Web-based portal—performance report.

The performance report includes outcome measurements, summary measurements, and the language sample data. SLPs can make data selections from programmed options (demonstrated in Figure 3), and the requested data items or language performance measurements from the selected time period will be shown in the performance report. According to the results of the questionnaire study, 91% (20/22) of the study participants believed that it would be important to know patients’ language performance while they are at home. Therefore, a checkbox is provided under each group of datasets so that SLPs can choose to review patients’ performance at home conveniently. From the selected data items or performance reports generated by the Web-based portal, clinicians can obtain a clear picture of their patients’ communication routines and progress over time and, therefore, design the corresponding AAC interventions for individual patients accordingly. For instance, SLPs can adjust their treatment materials and the mobile AAC app settings for each patient. If needed, SLPs can also obtain performance reports for a group of patients in different age groups with different types of communication disorders and determine whether these factors are associated with the patients’ activities using the mobile AAC app and their language outcome performance. The result may also be helpful for SLPs in adjusting their treatment strategy for patients.

**Figure 4:**
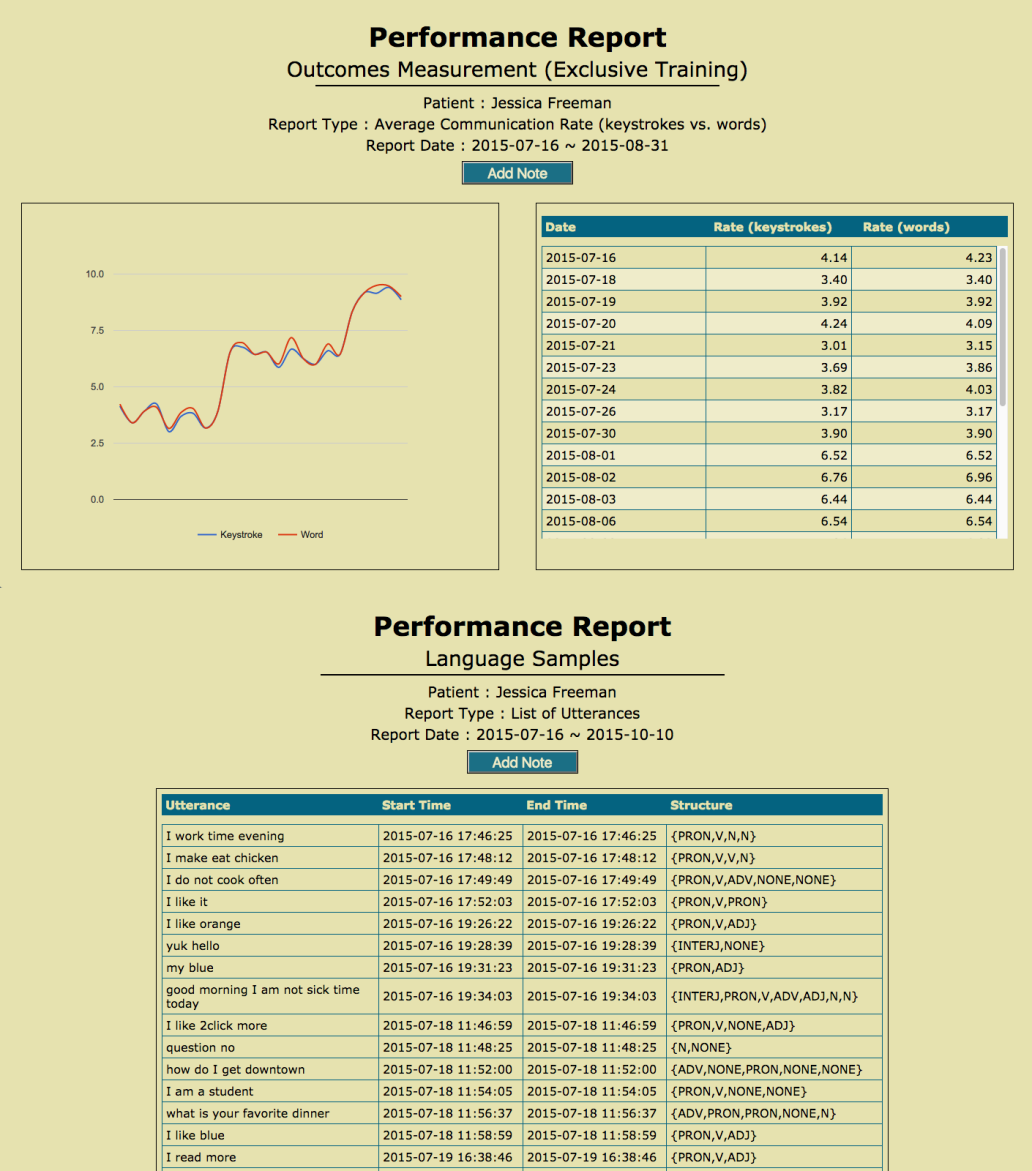
Web-based portal—performance report examples.

Figure 4 shows three examples of performance reports, each with a different set of information. First, the outcomes measurement shows daily average or total for each measure over the selected time period in a diagram and table (top portion of Figure 4). The summary measures provide an overall average or total of each measure over the selected time period (not shown). The language sample list (bottom portion of Figure 4) provides SLPs with detailed information on patients’ language performance. SLPs can choose to add notes to these performance reports after they review the data or results.

Besides the dashboard and performance reports, several other features have been implemented to support the automatic data analysis and AAC service delivery. These features include the following:

- *Appointments:* SLPs can use the appointment feature to track patients’ appointment dates.
- *Report Storage:* SLPs can easily save the generated performance reports for every therapy session with a few button clicks.
- *Raw Data Management:* SLPs can manually remove certain utterances from the analysis. This feature is especially useful if SLPs identify abnormal usage in the app. For instance, utterances generated in one particular time period may be far better than the ones typically generated by the patient. These utterances are most likely generated by caregivers for various purposes, and they should be removed when only patients’ language performance is desired.
- *Target Vocabulary Setting:* SLPs can set a list of target vocabulary for their patients, asking them to use those words frequently. After a certain period of time, SLPs can determine what percentage of this target vocabulary the patients have used. This target vocabulary can be easily updated on the Web-based portal.

### Evaluation on the mHealth Platform

A usability study was conducted with 20 AAC app users to evaluate the usability of the mobile app, and the study results were reported [21]. Another usability study was conducted with health care professionals to evaluate the usability of this Web-based portal while the data was generated in the mobile app. The health care professionals were also asked to compare the mHealth platform to the other two extant, abovementioned, data collection and reporting approaches. More specifically, professionals from the field of speech-language pathology were recruited for this study, including certified SLPs and graduate students in the Communication Science and Disorders program at the University of Pittsburgh. All participants were aged between 18 and 65 years. Ten individuals who met the inclusion criteria were selected from a pool of volunteers. They were recruited through a recruitment email script sent to the speech-language pathology communities or through word of mouth.

The study session commenced with a brief introduction to the study’s purpose and procedures. The participants were then asked to sign the informed consent form and fill out a questionnaire about background information such as age, years of clinical experience, years of computer experience, and other demographic information. Next, each participant was introduced to three different data collection and reporting approaches on AAC technologies, including our new platform. The participants were then asked to finish multiple tasks in data collection, analysis, and language performance report generation, and answer two “after-scenario” questions, adopted from the IBM “After Scenario Questionnaire” (ASQ) [22], wherein they utilized each of these three approaches. Upon completion of all assigned tasks, the participants were asked to complete a poststudy usability questionnaire and express their overall perception and level of satisfaction with the new mHealth platform. All activities during the hands-on session were logged and then analyzed afterward in order to evaluate the proposed approach. The participants were asked to review the three approaches and complete a poststudy questionnaire to indicate their respective preference for the three data collection and reporting approaches. Further feedback was collected from the participants in an informal interview session.

## Results

### Results of Usability Study

A total of 10 participants were recruited to participate in this usability study, including 5 SLPs and 5 graduate students from the Department of Communication Science and Disorders at the University of Pittsburgh. All participants were able to complete the assigned tasks and provide feedback through the questionnaires. Participants were aged between 22 and 61 years, mean age: 29.0 (SD 13.20) years. Of all, 80% participants were females and 20% were males. The participants’ work experience in AAC clinical services ranged from 0.5 to 30 years, mean experience: 4.8 (SD 8.64) years.

Two statements were chosen and modified from ASQ:

- Statement 1 (S1, easiness): “Overall, I am satisfied with the ease of completing this task.”
- Statement 2 (S2, efficiency): “Overall, I am satisfied with the amount of time it took to complete this task”.

All 10 participants responded to these statements on a 5-point Likert scale, where 1 corresponded to “strongly disagree” and 5 to “strongly agree.” In other words, a higher number indicated a greater satisfaction with the Web-based portal. Table 2 shows a breakdown of the ratings after each task. From these numbers, one can notice that it was easy and efficient for health care professionals to manage patients on this Web-based portal, with an average Likert rating of 4.7 on both statements after this task. They also indicated that reviewing the dashboard was easy and efficient, reporting a 4.4 on both statements. Apparently, it was slightly difficult to generate and review performance reports, however, with scores of 4.4 for easiness and 4.2 for efficiency. Some participants provided relatively low ratings regarding management of their patients’ profiles, especially for managing their language data, because that still needs to be handled manually. Overall, the app was rated as easy to use and efficient, evidenced by an overall score of 4.28.

The IBM Post Study System Usability Questionnaire (PSSUQ) was adopted to measure the Web-based clinician portal’s overall usability [22]. Fourteen statements were chosen from the PSSUQ and were slightly modified to include the phrase “the Web-based portal” instead of “the system.” All 10 participants completed this modified PSSUQ questionnaire. Participants responded to all 14 statements on a 5-point Likert scale, where 1 corresponded to “strongly disagree” and 5 to “strongly agree.” The overall average on all the usability factors was 4.18, which indicated satisfaction with this Web-based clinician portal. Table 3 presents a breakdown of the numerical ratings for each question. According to the conversations with participants at the end of the usability study, some of the lower ratings were assigned because the study session was their first time using the Web-based portal, and, thus, there was a learning curve associated with locating relevant information on the Web-based portal.

### User’s Preferred Approach

In the evaluation study, the participants were asked to compare our integrated mHealth platform with two extant data collection and reporting approaches. They were also asked to indicate their preference when choosing a data collection and reporting approach for use in their own clinical practice. Three more questions were created to collect the study participants’ opinions on the five key characteristics for a desired data collection and reporting system identified in the interview and questionnaire studies in AAC (comprehensive, automatic, real time, clinically relevant, and easily accessible). In sum, the results show that the participants agreed that our integrated mHealth platform could better support data collection and reporting in AAC service delivery. Table 4 shows the responses from the participants.

**Table 2:** Results from after-scenario tasks on the Web-based portal for statements 1 and 2 (S1 and S2; overall average=4.28).

Tasks and After Scenario Questionnaire	S1, mean (SD)	S2, mean (SD)
Task 1: Manage patient list	4.7 (.48)	4.7 (.48)
Task 2: Review dashboard	4.4 (.70)	4.4 (.70)
Task 3: Generate and review performance report	4.4 (.52)	4.2 (.42)
Task 4: Manage patient profile—language data	3.9 (1.20)	4.0 (.94)
Task 5: Manage patient profile—patient records	4.0 (.94)	4.1 (.74)

**Table 3:** The results of posttask usability study.

Item	Mean (SD)
Overall, I am satisfied with how easy it is to use this Web-based portal.	4.4 (.52)
It was simple to use this Web-based portal.	4.3 (.48)
I could effectively complete the tasks using this Web-based portal.	4.4 (.52)
I was able to complete the tasks quickly using this Web-based portal.	4.0 (.47)
I was able to efficiently complete the tasks using this Web-based portal.	4.5 (.53)
I felt comfortable using this Web-based portal.	3.8 (.63)
I believe I could become productive quickly using this Web-based portal.	4.5 (.53)
Whenever I made a mistake, I could recover easily and quickly.	4.0 (.67)
It was easy to find the information I needed.	3.8 (.79)
The organization of information on this Web-based portal was clear.	3.9 (.88)
The interface of this Web-based portal was pleasant.	4.1 (.32)
I liked using the interface of this system.	4.2 (.42)
This Web-based portal has all the functions and capabilities I expect it to have.	4.3 (.67)
Overall, I am satisfied with this system.	4.3 (.48)

**Table 4:** Comparison of three data collection and reporting approaches (N=10).

Questions	Mobile AAC^a^ app alone, n (%)	Integrated mHealth platform, n (%)	Dedicated AAC device, n (%)
Which approach collects the most comprehensive data?	0 (0)	9 (90)	1 (10)
Which approach provides the most automatic data collection and reporting?	0 (0)	10 (100)	0 (0)
Which approach provides the easiest access to the clinically relevant information you need?	2 (20)	7 (70)	1 (10)
Which of the approaches would you prefer to use?	0 (0)	9 (90)	1 (10)

^a^AAC: Augmentative and Alternative Communication.

Overall, 90% of the participants selected the new integrated mHealth platform as the approach they would prefer to use in their clinical services, while the other 10% chose the dedicated AAC device as the preferred approach. One possible reason for this 10% response is that dedicated AAC devices are currently the most widely used and many SLPs are familiar with the procedure, even though it is hard to obtain the desired data. Furthermore, 90% of the participants indicated that the integrated mHealth platform collects the most comprehensive data; 70% of participants believed that the integrated mHealth platform provides the easiest access to clinically relevant information. Notably, the participants did not believe that mobile AAC apps or dedicated AAC devices alone could provide automatic data collection and reporting. Markedly, 100% of participants believed that this integrated mHealth platform could provide the most automatic data collection and reporting.

### Results From Semistructured Interviews

Overall, the participants thought that the Web-based portal provided the most comprehensive information in the most efficient manner and that the user interface was both simple and intuitive. The data from dedicated AAC devices also appeared to be comprehensive, but the interface was too busy and the segmentation of utterances was labor-intensive. For the mobile AAC apps, the detailed utterance data was lost when the performance report was emailed since only the summary was included in the email. The following is some feedback from the participants:

Quickest, easiest, and nicest to look at, user friendly, easy to teach, nice graphic.

Participant 1

Data is always available on the clinician portal. The data was clearly divided into categories and was very comprehensive. The ability to search for specific time periods was very helpful.

Participant 5

I think that the Web-based portal provided the most comprehensive information in the most efficient manner. I also thought that the user interface was simple and intuitive. The program for analyzing the text file from the dedicated device also appeared to be comprehensive, but the interface was too busy and the selection of utterances seems labor-intensive. For the existing mobile AAC app, I didn't like that the detailed utterance data was lost when the performance report was emailed.

Participant 7

Web-based clinical portal is easy to use, data is collected in real time, and clinicians have access to information at any time.

Participant 10

Provides all date needed for intervention purposes.

Participant 4

Ability to gather data before the patient’s therapy session.

Participant 9

## Discussion

### Principal Results

In this project, we first conducted a mixed-method study, including a literature review, clinician interviews, and clinician surveys, to design the system architecture and identify design specifications, more specifically, the clinician-desired features and clinically relevant data items. Based on these findings, we accordingly created a mobile AAC app and a Web-based portal for SLPs. The Web-based portal makes it convenient for SLPs to track their patients’ situation in real time, which may be helpful for incorporating clinically relevant information into their clinical decision making and for designing personalized interventions. This may eventually lead to higher health care quality and lower costs.

After the Web-based portal was created, it was evaluated through a usability study with health care professionals. Participants were satisfied with the ease of completing all the tasks, such as viewing reports and managing patient data, as well as with the amount of time taken to complete each task. The overall results confirmed that our integrated mHealth platform provides the ability to collect comprehensive clinical evidence, automatically analyze collected data in real time, and generate clinically relevant performance measures. Our evaluation showed that the integrated platform offers a better clinical data analytics approach for AAC clinical service.

The research-based design of the integrated mHealth platform strongly supports its capabilities and application as an AAC tool. The features in the app provide PwCDs with an opportunity to efficiently and conveniently use the app as an AAC intervention tool; the features in the Web-based portal provide SLPs with an effective supporting tool for data collection and reporting. Since the rehabilitation process is usually long and sometimes frustrating, the performance reports can not only help SLPs to design appropriate treatment plans but also help PwCDs to gain confidence, which fulfills the desired outcomes of an evidence-based practice. Moreover, the clinically relevant data can benefit researchers in the field of communication science. Our mHealth platform can improve user engagement, as well as help SLPs adjust the treatment plans, support their clinical evaluation, and, ultimately, streamline the workflow and improve service delivery.

### Limitations

The study has some known limitations. First, for the evaluation of the Web-based portal, the study participants included were health care professionals. Patients were not included in the study, and were not using the mobile AAC app to provide real-time data. The study was conducted with a set of simulated data. In other words, the study participants did not see the dynamic changes in the datasets during the study and did not experience the power of real-time data collection and reporting, which could make the mHealth platform even more impressive. On the other hand, this limitation does not impact the results of this study. Since the goal of this study was to determine the usability of the mHealth platform, especially the Web-based portal, it does not matter whether the data was entered by patients at the time of the study. Second, when the study participants were asked to compare the three data collection and reporting approaches, they were only asked to indicate the preferred one in various circumstances. Therefore, preferences were only shown as percentages. Asking the participants to provide a rating on the characteristics of the three approaches would have been better. If that were the case, we would be able to have a better idea about participant preferences and about how much better this mHealth platform is compared with the other two approaches.

### Future Work

The work presented in this article is the foundation of our future work. This work demonstrated that the mHealth platform was well designed and implemented according to the needs of clinicians. It can be used to streamline the clinical data collection, analysis, and reporting. The availability of this mHealth platform significantly reduces the burden of clinicians regarding tedious and labor-intensive tasks. In the next step, to evaluate whether this mHealth platform can produce positive impact on the outcome of language rehabilitation, future research is being planned to conduct a larger-scale study, which will seek to evaluate all capabilities of the integrated mHealth platform. The proposed clinical trial will include both PwCDs and SLPs. A full AAC treatment will be provided from SLPs to PwCDs over a longer time period. During the study period, PwCDs will use the new mobile AAC app (EuTalk), while SLPs will periodically review their performance through the Web-based portal. SLPs will be asked to determine whether or not they need to adjust their treatment plans based on the performance reports provided in the Web-based portal. In this study, PwCDs’ clinical performance will be evaluated and SLPs will also be asked to evaluate the performance measures provided on the Web-based portal. Furthermore, the platform will be extended for all clinicians to easily access a summary of patient health data that is collected from multiple mHealth apps. The platform will be improved using sophisticated data analysis and data integration algorithms. The proposed enhanced mHealth data integration platform will enable customized, precise health care.

## Abbreviations

AAC: Augmentative and Alternative Communication
ASQ: After Scenario Questionnaire
IOM: Institute of Medicine
mHealth: mobile health
PSSUQ: Post Study System Usability Questionnaire
PwCD: person with communication disabilities
SLP: speech-language pathologist

## References

[1] Institute of Medicine (2013). Best Care at Lower Cost: The Path to Continuously Learning Health Care in America.

[2] Beiwinkel T, Kindermann S, Maier A, Kerl C, Moock J, Barbian G, Rössler W (2016). Using Smartphones to Monitor Bipolar Disorder Symptoms: A Pilot Study. JMIR Ment Health.

[3] Blake H (2008). Mobile phone technology in chronic disease management. Nurs Stand.

[4] Burke LE, Styn MA, Sereika SM, Conroy MB, Ye L, Glanz K, Sevick MA, Ewing LJ (2012). Using mHealth technology to enhance self-monitoring for weight loss: a randomized trial. Am J Prev Med.

[5] Goyal S, Morita P, Lewis GF, Yu C, Seto E, Cafazzo JA (2016). The Systematic Design of a Behavioural Mobile Health Application for the Self-Management of Type 2 Diabetes. Can J Diabetes.

[6] Hardinge M, Rutter H, Velardo C, Shah SA, Williams V, Tarassenko L, Farmer A (2015). Using a mobile health application to support self-management in chronic obstructive pulmonary disease: a six-month cohort study. BMC Med Inform Decis Mak.

[7] Kock A, Kaya R, Müller C, Andersen B, Langer T, Ingenerf J (2015). Design, implementation, and evaluation of a mobile application for patient empowerment and management of long-term follow-up after childhood cancer. Klin Padiatr.

[8] Pan D, Dhall R, Lieberman A, Petitti DB (2015). A mobile cloud-based Parkinson's disease assessment system for home-based monitoring. JMIR Mhealth Uhealth.

[9] Pramana G, Parmanto B, Kendall PC, Silk JS (2014). The SmartCAT: an m-health platform for ecological momentary intervention in child anxiety treatment. Telemed J E Health.

[10] Parmanto B, Pramana G, Yu DX, Fairman AD, Dicianno BE (2015). Development of mHealth system for supporting self-management and remote consultation of skincare. BMC Med Inform Decis Mak.

[11] Tregarthen JP, Lock J, Darcy AM (2015). Development of a smartphone application for eating disorder self-monitoring. Int J Eat Disord.

[12] Watanabe N, Horikoshi M, Yamada M, Shimodera S, Akechi T, Miki K, Inagaki M, Yonemoto N, Imai H, Tajika A, Ogawa Y, Takeshima N, Hayasaka Y, Furukawa TA, Steering Committee of the Fun to Learn to ActThink through Technology Project (2015). Adding smartphone-based cognitive-behavior therapy to pharmacotherapy for major depression (FLATT project): study protocol for a randomized controlled trial. Trials.

[13] Gay V, Leijdekkers P (2015). Bringing Health and Fitness Data Together for Connected Health Care: Mobile Apps as Enablers of Interoperability. J Med Internet Res.

[14] Raja A, Tridane A, Gaffar A, Lindquist T, Pribadi K (2014). Android and ODK based data collection framework to aid in epidemiological analysis. Online J Public Health Inform.

[15] Morris MA, Meier SK, Griffin JM, Branda ME, Phelan SM (2016). Prevalence and etiologies of adult communication disabilities in the United States: Results from the 2012 National Health Interview Survey. Disabil Health J.

[16] Dollaghan C (2007). A Handbook for Evidence-Based Practice in Communication Disorders.

[17] Hill K, Baker B, Romich B, Cooper RA, Ohnabe H, Hobson DA (2007). Augmentative and Alternative Communication Technology. An Introduction to Rehabilitation Engineering.

[18] Hill K (2001). The Development of a Model for Automated Performance Measurement and the Establishment of Performance Indices for Augmented Communicators Under Two Sampling Conditions, University of Pittsburgh.

[19] Hershberger D (2011). Mobile Technology and AAC Apps From an AAC Developer's Perspective. Perspect Augment Altern Commun.

[20] Wang E, Zhou L, Parmanto B, Watzlaf V, Abdelhak M (2018). Clinician's Perceptions and Expectations on a mHealth Platform for Supporting Patient Data Integration and Clinical Service Delivery: A Case Study in Evidence-Based Communication Rehabilitation.

[21] Wang E, Zhou L, Chen SK, Hill K, Parmanto B (2017). Development and evaluation of a mobile AAC: a virtual therapist and speech assistant for people with communication disabilities. Disabil Rehabil Assist Technol.

[22] Lewis JR (1995). IBM computer usability satisfaction questionnaires: Psychometric evaluation and instructions for use. International Journal of Human-Computer Interaction.

